# Do Insomnia Treatments Improve Daytime Function?

**DOI:** 10.3390/jcm12093089

**Published:** 2023-04-24

**Authors:** Nathaniel F. Watson, Suzanne M. Bertisch, Charles M. Morin, Rafael Pelayo, John W. Winkelman, Phyllis C. Zee, Andrew D. Krystal

**Affiliations:** 1 Department of Neurology, University of Washington School of Medicine, Seattle, WA 98104, USA; 2 Department of Medicine, Brigham and Women’s Hospital, Harvard Medical School, Boston, MA 02130, USA; 3 Department of Psychology, Cervo/Brain Research Centre, Laval University, Québec City, QC G1J 2G3, Canada; 4 Department of Psychiatry and Behavioral Sciences, Stanford University Sleep Medicine Center, Redwood City, CA 94063, USA; 5 Department of Psychiatry, Massachusetts General Hospital, Boston, MA 02114, USA; 6 Department of Neurology, Massachusetts General Hospital, Boston, MA 02114, USA; 7 Department of Neurology, Center for Circadian and Sleep Medicine, Northwestern University, Evanston, IL 60209, USA; 8 Department of Psychiatry, UCSF Weill Institute for Neurosciences, San Francisco, CA 94103, USA; 9 Department of Neurology, UCSF Weill Institute for Neurosciences, San Francisco, CA 94103, USA

**Keywords:** insomnia, medications, daytime function, clinical appraisal

## Abstract

A scientific advisory panel of seven U.S. and Canadian sleep experts performed a clinical appraisal by comparing general medical opinion, assessed via a survey of practicing clinicians, regarding insomnia treatment, with the available scientific evidence. This clinical appraisal focuses on the specific statement, *“Treatments for insomnia have uniformly been shown to significantly improve the associated daytime impairment seen with insomnia.”* The advisory panel reviewed and discussed the available body of evidence within the published medical literature to determine what discrepancies may exist between the currently published evidence base and general medical opinion. The advisory panels’ evaluation of this statement was also compared with the results of a national survey of primary care physicians, psychiatrists, nurse practitioners, physician assistants, and sleep specialists in the United States. Contrary to general medical opinion, the expert advisory panel concluded that the medical literature did not support the statement. This gap highlights the need to educate the general medical community regarding insomnia treatment efficacy in pursuit of improved treatment outcomes.

## 1. Introduction

Sleep is a crucial physiological function considered as important as diet and exercise to human health and wellbeing [1,2]. According to the Centers for Disease Control and Prevention, sleep disorders affect about 30% of the general population in the U.S. [3]. Insomnia is the most common sleep disorder and can have devastating effects on human health, including a higher risk of cardiovascular disease, alcohol abuse, and incident and recurrent depression [4,5]. Sleep disruption also affects daytime function; it is associated with increased risk of automotive and other accidents, poor work performance, work absenteeism, and social isolation [6,7].

Insomnia identification and management is complex and evolving. Network visualization techniques offer novel methods to generate terminology databases to facilitate insomnia identification [8]. Several new pharmacologic treatments have come forth, expanding therapeutic options. Yet, despite the availability of numerous different pharmacologic therapies with novel mechanisms of action, optimal treatments for specific insomnia symptoms, such as daytime impairment, are not well-defined or standardized [9,10].

Insomnia is also a clinically heterogeneous disease. Patients may have difficulty falling or staying asleep, wake too early, or have physical or psychological factors complicating their insomnia symptoms. They may experience daytime impairment or limited disability due to their condition. Although the definition of insomnia includes both daytime and nighttime symptoms, symptoms of daytime impairment may not be fully considered when evaluating patients and assessing treatment outcomes. Indeed, despite daytime impairment being part of insomnia diagnostic criteria, the U.S. Food and Drug Administration (FDA) does not require improvement of daytime impairment for medication approval. As a result, the effects of insomnia treatment on daytime function are less systematically assessed in treatment trials and less consistently obtained than effects on indices of nighttime sleep. These factors may predispose towards a general mindset amongst prescribing providers that daytime symptoms should improve with treatment simply by virtue of improvement in nighttime aspects of insomnia. Challenging this assumption through examination of the relevant clinical literature forms the basis of this clinical appraisal.

We must acknowledge that modern medicine, with all its complexity and demands, provides limited opportunities for providers to always be knowledgeable about the latest therapeutic trends. Indeed, the average clinical provider has limited training in sleep medicine best practices. Yet, these providers must naturally strive to pair the best therapy with the unique needs of each individual patient. Providers endeavor to make decisions based on the best available data, but this does not always reflect a critical appraisal of all available evidence.

In carrying out this clinical appraisal, we considered a broad array of insomnia treatments; both pharmacological and non-pharmacological. Cognitive behavioral therapy for insomnia (CBT-I) is a non-pharmacological therapy often considered first-line treatment for insomnia. CBT-I is a multicomponent intervention combining behavioral and cognitive techniques to reduce cognitive arousal and enhance sleep regulation [11]. Although effective, CBT-I may be inaccessible for many patients due to the limited availability of trained providers.

Pharmacological insomnia therapies approved by the U.S. FDA for treatment of insomnia fall into four categories: GABA-A-receptor modulators, melatonin receptor agonists, selective antihistamines, and selective dual orexin receptor antagonists (DORAs). In addition, drugs originally FDA-approved for treatment of depression (e.g., trazodone, amitriptyline, etc.), psychosis (e.g., quetiapine, risperidone, olanzapine), seizure disorders (e.g., gabapentin, pregabalin, etc.), or hypertension (e.g., prazosin, clonidine) are sometimes used “off-label” to treat insomnia but generally have little to no evidence base related to their use in treating insomnia. The lack of a robust evidence base also pertains to insomnia treatments taken over the counter (OTC), most of which are herbals (e.g., valerian, kava-kava) or contain diphenhydramine, chlorpheniramine, or doxylamine. Note that melatonin is OTC, while melatonin agonists require a prescription. Summarized below are the classes of FDA-approved insomnia medications:

- GABA (Gamma-aminobutyric acid) is the most common inhibitory neurotransmitter in the brain. Often used to treat insomnia, GABA-A receptor modulators (benzodiazepines and so-called “non-benzodiazepines”: zolpidem, zaleplon, eszopiclone, zopiclone) act by binding to GABA-A receptors in the brain and thereby enhance the inhibition of nerve cell activity. The resulting effects can include sleep enhancement, anxiety reduction, anti-seizure effects, and psychomotor impairment. Insomnia therapies in this category may have abuse liability and untoward side effects such as next-day drowsiness or increased fall risk.
- Melatonin agonists include the selective melatonin agonist ramelteon, which agonizes MT1 and MT2 receptors producing sleep onset-enhancing effects without sleep maintenance effects. Abuse liability is absent and adverse effects are minimal. The hormone melatonin is also a melatonin agonist. It is available over the counter, but the published literature suggests its effects on sleep are not clinically significant in insomnia patients.
- Selective histamine H1 antagonists block the wake-promoting effects of the neurotransmitter histamine. The only such agent FDA-approved for insomnia, doxepin dosed at 3–6 mg, has effects on sleep maintenance, including the last third of the night, but without robust effects on sleep onset. Further, this agent is without abuse liability and has few side effects in terms of having very limited effects other than sleep enhancement.
- Dual orexin receptor antagonists (DORAs) are the newest class of drugs FDA-approved to treat insomnia. These medications block the effects of the neuropeptide orexin which functions to promote alertness and wakefulness. Three DORA drugs, suvorexant, lemborexant, and daridorexant, are currently FDA-approved for chronic insomnia. These agents have robust effects on both sleep onset and maintenance and, like doxepin 3–6 mg, are unique in having therapeutic effects in the last third of the night. Although they have some abuse liability, it is limited compared with GABA-A modulators and they have relatively few side effects beyond sleep enhancement.

Clinical appraisal meetings seek to address potential limitations in clinical management due to gaps in practitioner knowledge of the available literature. These appraisals compare general clinical practice to optimal evidence-based clinical practice to assess if gaps exist. They provide a platform from which world leaders in a given medical discipline can determine the best way forward to ensure both medical knowledge and real-world disease management are kept up to date. This manuscript presents the results of one of the primary focal points of an insomnia-centered clinical appraisal meeting held in Chicago, Illinois in December 2021 by specifically addressing the statement, “*Treatments for insomnia have uniformly been shown to significantly improve the associated daytime impairment seen with insomnia*”.

## 2. Materials and Methods

### 2.1. Field Survey and Appraisal Design

A scientific advisory panel of U.S. and Canadian-based sleep medicine experts directed these meetings (see manuscript author list). The advisory panel first developed seven statements considered most relevant to current insomnia therapy—where gaps may exist between clinical practice habits and the published medical literature. These statements concerned the pros and cons of different treatment methods and included issues of dependency, addiction, daytime impairment, and the uniformity of clinical effects within drug or treatment classes. A comprehensive national online survey of clinical practitioners then focused on the seven predetermined statements to gather formative data on current practice patterns to inform potential knowledge gaps relevant to insomnia therapy. 

Concurrent with the online survey, the advisory panel reviewed the published literature both in support of, and refuting, the seven statements. For all statements, a formal search of the literature was performed, which included, but was not limited to, review of current clinical guidelines, review articles, and clinical trials. The panel then met to present their findings to their peers for appraisal and discussion, and to review and discuss how their expert opinion compared to survey-determined current practice trends. This manuscript addresses the predetermined statement relating to daytime function and discusses the findings of the scientific advisory panel concerning the focus statement, “*Treatments for insomnia have uniformly been shown to significantly improve the associated daytime impairment seen with insomnia*”.

A 10-minute online survey was sent to health care professionals from specialties including primary care providers (PCPs)/family practice, psychiatrists, and sleep specialists across the United States. The survey included seven predetermined statements, launched in two waves: wave one (December 2021): *n* = 108 and wave two (April 2022): *n* = 400. Survey recipients were asked to what degree they supported or rejected each statement. Of the 508 total eligible respondents, the breakdown subspecialty is as follows: MDs (74%), nurse practitioners (14%), physician assistants (11%) and PhD (1%). Survey respondents were also given the option of providing reasoning for their votes on the statement. The survey respondents voted on their level of acceptance or rejection of the statement based on a Likert scale. Support levels are shown below and were defined on a scale of 1 to 6 with 6 representing complete rejection of the statement.

Survey criteria: Level of Support for the Statement

1. Strongly agree
2. Mostly agree, but with minor reservations
3. Slightly agree, but with major reservations
4. Slightly disagree due to minor reservations
5. Mostly disagree due to major reservations
6. Strongly disagree

Concurrent with the first wave of the field survey, each member of the expert advisory panel assessed each of the seven statements. Each panel member then chose a statement on which to conduct an in-depth literature review of the published research and clinical evidence pertinent to that statement. Subsequently, the panel of seven subject matter experts gathered in Chicago, Illinois in December 2021 to present the evidence for and/or against each statement. After reviewing and discussing the data, panel members rated each statement again. The panel also ranked the overall quality of the evidence presented, which included the following categories:

1. Evidence obtained from meta-analysis, including at least 1 large, randomized controlled trial (RCT)
2. Evidence obtained from either meta-analysis, including at least 1 small RCT or from at least 1 well-designed, large RCT
3. Evidence obtained from well-designed cohort or case-controlled studies
4. Evidence obtained from case series, case reports, or flawed clinical trials
5. Opinions of respected authorities based on clinical experience, descriptive studies, or reports of expert committees
6. Insufficient evidence to form an opinion

**Figure 1:**
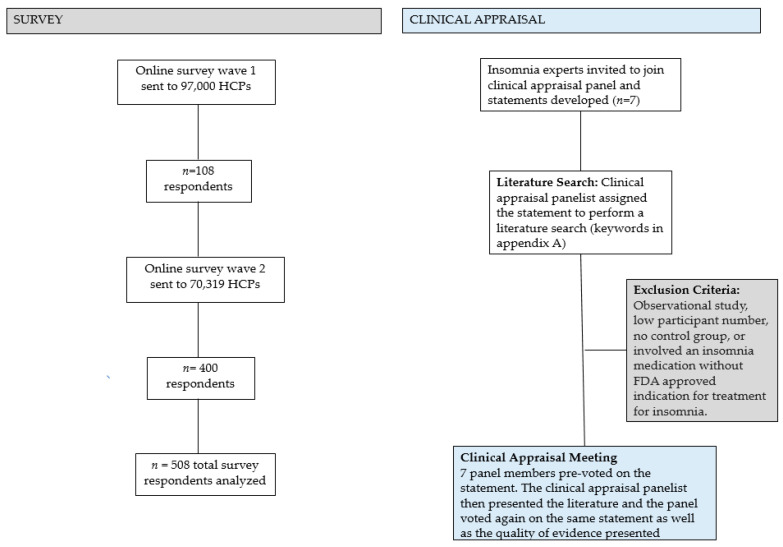
Summary of the survey and clinical appraisal panel methodology. Eighteen articles were selected for presentation. HCP = health care provider.

RCTs containing >1000 patients were deemed “large”. Small to moderate trial sizes were defined as <50 per group. We eliminated inferior studies (observational studies, very low participant number, no control group, inherent bias) during the literature search process. The results of this clinical appraisal are presented in this manuscript and will be used as a framework to guide future treatment recommendations. Please see Figure 1 for a summary of the survey and clinical appraisal methodology. 

### 2.2. Literature Search Methodology

A literature search was performed in October 2021 using the PubMed search engine assessing the MEDLINE database. Searches were limited to humans and in English. To identify trials not registered in electronic databases, we contacted key informants and searched reference lists of identified studies. We also searched the Cochrane Central Register of Controlled trials (CENTRAL), MEDLINE, Embase, PsycINFO, PSYNDEX and registry databases (WHO trials portal, ClinicalTrials.gov, accessed on 12 November 2021) with results incorporated from searches to 10 February 2022. Specific search terms are specified in Appendix A.

## 3. Results

### 3.1. Evidence Review

#### 3.1.1. Evidence in Support of the Focus Statement

After carrying out an in-depth literature review based on the search criteria specified above, a panel member (NFW) presented an overview of the scientific evidence most closely related to their chosen focus statement. Whilst it was noted that the majority of studies in the literature do not specifically address daytime impairment, there was only one set of studies that utilized a validated assessment of daytime function (IDSIQ) as a prespecified endpoint. Nonetheless, summarized below are the studies supporting the focus statement.

1. GABA-A Modulators:

Eszopiclone

Clinical studies examining daytime impairment during treatment with eszopiclone support the focus statement. A study by Liang et al. published in 2019 described a systematic review and meta-analysis of double-blind, randomized, placebo-controlled clinical trials to evaluate the efficacy and safety of eszopiclone for the treatment of primary insomnia [12]. The analysis included a total of six randomized trials involving 2809 patients with primary insomnia. Eszopiclone treatment was associated with increased subjective sleep quality, ability to function, daytime alertness and sense of physical well-being at 1 week, 1 month, 3 months and 6 months.

A publication by Rosner et al. included the description of 14 parallel group RCTs comparing eszopiclone with either a placebo or active control [13]. Participants included a total of 4732 adults with insomnia, diagnosed via a standardized system, and included individuals with both primary and comorbid insomnia. The trials covered short-term (≤4 weeks; 6 studies), medium-term (>4 weeks ≤6 months; 6 studies) and long-term treatment (>6 months; 2 studies) with eszopiclone. Mean next day alertness, as indicated on a 0–10 Likert scale, in the intervention group was 0.46 points higher (95% CI: 0.28 to 0.63 higher) than the control group, indicating a positive effect of eszopiclone on this daytime measure.

2. Dual Orexin Receptor Antagonists (DORAs):

Suvorexant

Two clinical studies on suvorexant treatment support the focus statement. One publication presented data pooled from two similar Phase 3, randomized, double blind, placebo-controlled, parallel group, 3-month trials of the DORA drug suvorexant [14]. Study participants received suvorexant administered at dose regimes of either 20/15 mg, 40/30 mg, or placebo. Trial participants completed a seven-item Insomnia Severity Index (ISI) questionnaire at baseline and after months 1 and 3, indicating their perception of their sleep disorder on a scale of (0 = none to 4 = very severe). ISI items 5, 6, and 7, which all address daytime symptoms, were tallied to generate an “impact” score:

Item 5: Sleep problem interferes with daily function

Item 6: Sleep problem impairs quality of life

Item 7: Worried/distressed about sleep problem 

Impact is 5 + 6 + 7

ISI total scores and impact scores were less from Month 1 to Month 3 in both suvorexant groups and the placebo group relative to baseline. The mean changes in scores on most of the individual ISI items were also improved to a greater extent in the suvorexant groups than the placebo group, with the strongest effects tending to be seen on the “dissatisfied with current sleep pattern” item.

These findings regarding suvorexant are similar to those from an earlier study reported by the same group [15]. In 2016, the group presented the results from two randomized, double-blind, placebo-controlled, parallel 3-month trials in nonelderly (18–64 years) and elderly (≥65 years) patients with insomnia where they assessed aspects of daytime functioning. The study included 1021 subjects in trial 1 and 1009 in trial 2. Suvorexant doses of 40/30 mg (nonelderly/elderly) and 20/15 mg (nonelderly/elderly) were evaluated. ISI data and data from the six-point Patient Global Impression-Severity scale (PGI-I) were collected to monitor daytime impairment. Improvements with suvorexant at a 20/15 mg dosage were apparent on patient- and physician-rated global outcomes at all time points. The PGI-I scale also includes sleep related measures, so these particular results are not specifically focused on daytime symptoms. 

Daridorexant

In the only studies to utilize a validated assessment to gauge daytime function, the first of which was multicenter, phase-3, double-blind, placebo-controlled, parallel-group study, 930 adult and elderly patients with insomnia were randomized (1:1:1) to placebo or daridorexant at 25 mg or 50 mg, taken nightly for 3 months, after a placebo run-in and followed by a week-long placebo run-out [16,17]. Secondary endpoints included daytime function using the sleepiness domain of the validated Insomnia Daytime Symptoms and Impacts Questionnaire (IDSIQ) at 1 month and 3 months. Daridorexant at 50 mg significantly improved IDSIQ sleepiness domain scores at 1 month and 3 months versus placebo (*p* < 0.0003). The 3-month result is also considered clinically significant. In an exploratory endpoint, daridorexant demonstrated improvement in daytime function from baseline at 1 month and 3 months for the IDSIQ total score and other individual domains (mood, cognition) for the 50 mg dose. For the 25 mg dose, improvement in the IDSIQ score at 1 month and 3 months vs. placebo was not statistically significant (sleepiness domain) and/or were not prespecified key endpoints (other domains).

3. Anti-depressants:

Trazodone

A 2011 study investigated whether the outcome of treatment with trazodone CR in primary insomnia differs between patients with and without subthreshold depression [18]. Fifteen patients with primary insomnia and low Beck Depression Inventory (BDI) scores, and 14 patients with primary insomnia and increased BDI scores, were treated with trazodone CR at 25–150 mg/d for 3 months and followed up for 1 month after discontinuation of the medication. Patients generally reported improvement in their sleep and daytime functioning on all items in the Leeds Sleep Evaluation Questionnaire (a tool widely used to evaluate sleep disorders), with comparable results reported in both groups. Treatment with trazodone CR significantly improved daytime functioning as measured with the Sheehan Disability Scale. This was true in patients both with and without subthreshold depression.

4. Non-pharmacological Therapies:

Cognitive Behavioral Therapy for Insomnia (CBT-I)

A systematic review published in 2017 examined the impact of CBT-I on daytime cognitive functioning. Eighteen studies met inclusion criteria showing evidence for small to moderate effects of CBT-I on subjective measures of cognitive functioning [19]. However, few of the effects were statistically significant, likely due to small sample sizes and limited statistical power. An additional meta-analysis of 86 studies was conducted in 2020, and concluded that CBT-I, by improving nighttime symptoms, has a small but positive effect on daytime symptoms, but does not target the daytime symptoms directly [20]. Lastly, a recent AASM systematic review and meta-analysis showed that three patient subgroups (insomnia with psychiatric comorbidities, insomnia with medical comorbidities, and insomnia with no comorbidities) met the clinical significance threshold for daytime fatigue improvements favoring the CBT-I treatment [21].

#### 3.1.2. Evidence in Support of and against the Focus Statement

In some cases, clinical studies reported both evidence that supported the focus statement, and evidence that did not support the statement.

1. GABA-A Modulators:

Zolpidem ER

Escitalopram, an anti-depressive and anti-anxiolytic drug in the SSRI class, was administered to 385 patients at 10 mg/day. Trial participants were then randomized to concomitant treatment with zolpidem extended release (ER) at 12.5 mg/night or placebo for 8-weeks in a randomized, parallel-group, multicenter trial [22]. Treatment with zolpidem ER/escitalopram was associated with statistically significant differences (*p* < 0.05) in the Sleep Impact Scale measuring energy versus fatigue, daily activities, emotional well-being, social well-being, and sleep satisfaction scores at 24 weeks compared to placebo/escitalopram treatment. No difference was seen for the mental fatigue domain. The treatment groups did not exhibit significant differences in any of the Massachusetts General Hospital Cognitive and Physical Functioning Questionnaire subscales at week 24, which includes wakefulness/alertness, energy, motivation and enthusiasm, attention and focus sustainability, memory and recall, ability to find words, and mental acuity. On 2 of 10 Quality of Life Enjoyment and Satisfaction Questionnaire subscales, the advantages of zolpidem ER/escitalopram treatment were significant (*p* < 0.05), whereas on one of the subscales (school/coursework), the advantage for placebo/escitalopram was significant (*p* < 0.05). Treatment with zolpidem ER/escitalopram resulted in significantly greater improvement on the Morning Sleep Questionnaire items energy and sleep impact on daily activities (both *p* values < 0.05) but not for morning concentration ability.

2. Dual Orexin Receptor Antagonists (DORAs):

Lemborexant

In 2020, Kärppä et al. reported the results of a 12-month, global, multicenter, randomized, placebo-controlled, and parallel group-controlled Phase 3 clinical trial of lemborexant [23]. The study included results from 949 adult patients with insomnia. Morning sleepiness and alertness were self-reported on a scale of 1–9. Mean changes from baseline in morning sleepiness/alertness scores at the end of month 6 were numerically greater (i.e., improved) with lemborexant treatment versus treatment with a placebo. This change was statistically significant with the lemborexant 10 mg treatment (*p* < 0.05) but not with the lemborexant 5 mg treatment, indicating participants tended to be more alert in the morning following treatment with lemborexant at the higher dosage, but not at the lower dosage, compared with a placebo.

Daridorexant

In a Phase 2 trial, 359 elderly (≥64 years) participants with insomnia were randomly allocated to receive one of six treatment regimens (placebo, 5, 10, 25, and 50 mg daridorexant, or 10 mg zolpidem) for 30 days [24]. The dosage regimen covered 5 treatment periods, each consisting of 2 treatment nights followed by a 5-day to 12-day washout period. The results of this trial did not establish a dose-dependent effect. Self-reported next-day performance as to morning sleepiness, daytime alertness, and daytime ability to function, were assessed by the visual analog scale (VAS) for sleep quality. VAS scores on morning sleepiness, daytime alertness, and daytime ability to function all showed nonsignificant increases in a dose-dependent manner at higher doses of daridorexant compared with the placebo at week 2, which was not sustained at week 4. Scores for the Digit Symbol Substitution Test (DSST; evaluating attention, perceptual, and motor speed) were higher than baseline and scores for the Karolinska Sleepiness Scale (KSS; next-day alertness) and Sheehan Disability Scale (SDS; impairment in work, social life, and home responsibilities) showed no dose-dependent effect.

In a similar, smaller clinical study on the effects of daridorexant in older adults with insomnia, 58 study participants (≥65 years) were randomly allocated to receive 5 treatments (consisting of 5, 10, 25, and 50 mg daridorexant or a placebo) during 5 treatment periods, each consisting of 2 treatment nights followed by a 5-day to 12-day washout period [16]. Participants self-reported next-day performance using VAS. Performance was numerically improved, but not statistically significant across all domains from baseline to days 1 and 2 in all groups. A relationship of improvement with daridorexant dose was observed only for morning sleepiness, up to 25 mg. Scores for the DSST were higher while the KSS and SDS were lower than baseline at days 1 and 2 (i.e., all showed improvements) in all treatment groups, with no differences between male and female participants. Changes from baseline in DSST, KSS, and SDS scores did not demonstrate a correlation with morning plasma concentrations of daridorexant.

#### 3.1.3. Evidence against the Focus Statement

1. GABA-A Modulators:

Eszopiclone, Zaleplon, Zolpidem, Temazepam, Triazolam, Midazolam, Estazolam, Brotizolam, Diazepam, Flurazepam, Chlordiazepoxide

Holbrook et al. reported results of a meta-analysis carried out to systematically review the benefits and risks associated with the use of benzodiazepines to treat insomnia in adults [25]. Research studies were included in the meta-analysis if they were randomized controlled trials involving patients with insomnia comparing treatment with a benzodiazepine to treatment with a placebo or another active agent. Forty-five trials met the criteria, representing a total of 2672 patients. Patients receiving benzodiazepines reported more adverse effects, especially daytime drowsiness, than the placebo group (odds ratio 1.8, 95% CI 1.4 to 2.4).

The FDA labeling information for non-benzodiazepine hypnotics (eszopiclone, zaleplon, and zolpidem) warns of daytime memory and psychomotor impairment, abnormal thinking, and behavioral changes. In an evidence report for a clinical practice guideline supporting this labeling, the authors found an association of benzodiazepine and non-benzodiazepine hypnotic use with fractures, daytime somnolence, and motor vehicle accidents [26].

2. Melatonin Receptor Agonists:

Ramelteon

Another study evaluated the effects of ramelteon on insomnia in patients with treated obstructive sleep apnea (OSA) [27]. This study was a parallel group, randomized, double-blind, placebo-controlled pilot effectiveness clinical trial. The trial enrolled 21 research study participants who were ≥60 years old and had OSA accompanied by complaints of insomnia. Research study participants, all of whom were starting on positive airway pressure machines for their sleep apnea, were randomized to ramelteon at 8 mg (*n* = 8) or a placebo (*n* = 13). No statistically significant changes in daytime functioning were noted when comparing ramelteon vs. placebo.

3. Anti-depressants:

Trazodone

A study evaluated treatment of 16 patients with insomnia using trazodone [28]. Trazodone at 50 mg was administered to participants 30 min before bedtime for seven days in a three-week, within-subjects, randomized, double-blind, placebo-controlled study. Subjective effects, equilibrium (anterior/posterior body sway), short-term memory, verbal learning, simulated driving, and muscle endurance were assessed the morning after Days 1 and 7 of drug administration. Sleep was evaluated with overnight polysomnography and modified Multiple Sleep Latency Tests (MSLT) on Days 1 and 7. Trazodone produced small but significant impairments of short-term memory, verbal learning, equilibrium, and arm muscle endurance across all time points.

#### 3.1.4. Grading of the Literature and Level of Statement Support

Literature presented in support or against the statement was evaluated by the seven panelists. The majority of panelists (86%) voted the evidence presented was obtained from meta-analysis, including at least one large, randomized, controlled trial or from either meta-analysis, including at least one small, randomized, controlled trial or from at least one well-designed, large, randomized controlled trial. This represents the two highest evidence ratings on the 1–6 scale presented previously in this manuscript (i.e., 1 and 2 on the scale of evidence presented).

Regarding the level of support for the focus statement: “*Treatments for insomnia have uniformly been shown to significantly improve the associated daytime impairment seen with insomnia*” the panel was asked to vote on their level of acceptance for the statement, after which the level of acceptance by field survey respondents was presented.

**Figure 2:**
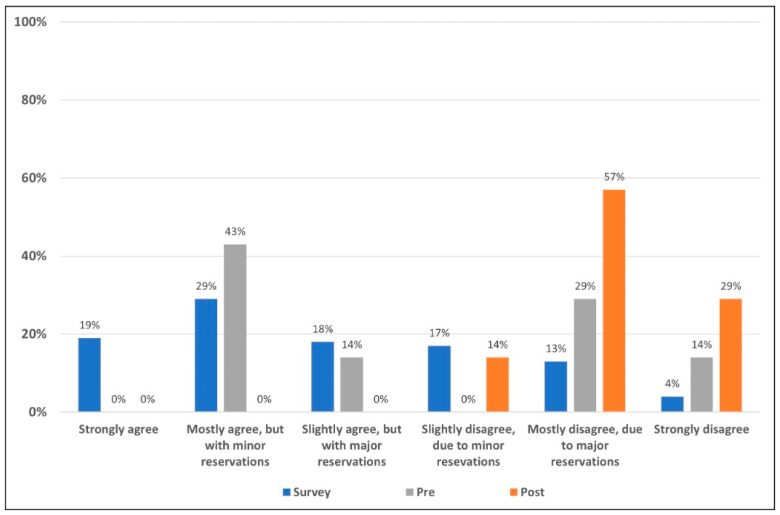
Level of acceptance/rejection for field survey respondents and the expert panel pre- and post-presentation of evidence. The 508 survey respondents voted on their level of acceptance or rejection of the statement based on a six-point Likert scale (blue). Prior to seeing field survey results, and discussing the literature, the seven members of the appraisal panel voted on their level of acceptance/rejection (gray). The literature and data supporting and refuting the statement were then reviewed, discussed and the same seven-member panel voted once more (orange) on their levels of acceptance/rejection using the same six-point Likert scale.

As illustrated in Figure 2, the majority of field survey respondents agreed with the statement to some extent. Prior to the presentation of evidence, panel members tended toward agreeing with the statement. Following the presentation of the literature and discussion of the presented evidence, the panelists were stronger in level of rejection of the statement as can be seen in Figure 2.

**Figure 3:**
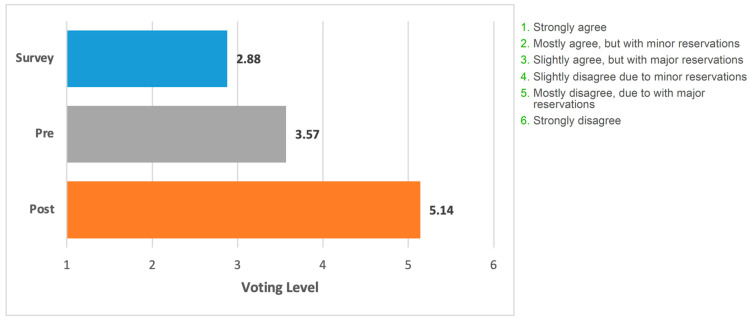
Mean voting level for the statement. Blue bar, field survey respondents (*n* = 508). Grey bar, appraisal panel (*n* = 7) before meeting. Orange bar, appraisal panel members (*n* = 7) after meeting and presentation of the literature. The grading on the *x*-axis corresponds to the levels of support/rejection of the statement.

Figure 3 shows voting data presented as an average of the level of acceptance/rejection for the focus statement. (i.e., levels 1–6).

## 4. Discussion

Our focus in this specific clinical appraisal concerned the statement, *“Treatments for insomnia have uniformly been shown to significantly improve the associated daytime impairment seen with insomnia.”* We reviewed the published literature related to this question and compared the perspective of experts reviewing this literature to the perspective of individuals managing insomnia in clinical practice. We found the majority of studies failed to report the effects of insomnia treatment on daytime function. Even in those studies that do specifically address daytime function, the nature and severity of daytime impairment is not well documented or defined. This is surprising given that daytime impairment is part of the diagnostic criteria for insomnia according to the latest versions of the American Psychiatric Association’s Diagnostic and Statistical Manual of Mental Disorders and the International Classification of Sleep Disorders [29]. This also represents a significant impediment to definitively resolving the focus statement of this appraisal.

Another challenge is that most insomnia medications have some degree of sleepiness or fatigue as a side effect. This occurs more frequently with some medications than others. This fact alone provides convincing evidence of the falsity of the statement that is the focus of this report. Another key aspect of the focus statement was the inclusion of the word “uniformly.” The body of clinical evidence clearly negates this aspect since some data supported the statement and some did not.

The results of the clinical appraisal field survey demonstrate the majority of prescribing physicians agree with the focus statement, i.e., that in general, all insomnia treatments significantly improve daytime impairment. Prior to the presentation of clinical data, our panel of sleep experts displayed a more mixed response, yet the majority still agreed with the focus statement despite having reservations. Following a review and discussion of the pertinent clinical research data, panel member’s views notably shifted. Voting results now showed the majority of the panel disagreed with the focus statement, revealing a discrepancy between prescribing physicians’ beliefs regarding treatment and actual clinical outcomes. The primary reason for this shift was evidence from multiple large clinical trials involving several different treatment options that did not support the idea of uniform improvement in daytime functioning. For example, trazodone treatment was assessed on several measures of daytime function and instead of leading to improvement, produced small but significant impairments of short-term memory, verbal learning, equilibrium, and arm muscle endurance across a series of timepoints. Administration of ramelteon did not improve daytime functioning across several assessment criteria. In contrast to these and other clinical studies, a number of agents were associated with significant improvements in measures of daytime function including the GABA-A modulator eszopiclone and the DORAs suvorexant and daridorexant. Evidence for significant improvement in daytime function was also reported for the 10 mg dosage of the DORA lemborexant. 

The expert panel determined that although the evidence volume was not as ample as hoped, the quality of the evidence addressing this question was high. Given the discrepancy between field survey results representing consensus medical opinion and clinical appraisal panel voting after the review of actual clinical evidence, the panel recognized an unmet need to address these gaps in knowledge. Future insomnia medication clinical trials would do well to include a measure of daytime functioning as a key primary or secondary outcome. The insomnia daytime symptoms impact questionnaire (IDSIQ) is a patient-reported outcome measure validated in insomnia patients and available for use in future insomnia medication clinical trials [30]. This publication represents part of the ongoing effort to educate and update primary care providers and others in the field regarding these study findings. We hope this work motivates providers to ask about daytime symptoms and functioning when assessing treatment efficacy in patients with insomnia.

One of the challenges with this particular focus statement is that daytime functioning was often not considered in insomnia research and clinical trials, and when it was, the measures were inconsistent, obviating comparative effectiveness between medications. Comprehensive inclusion of endpoints including daytime functioning is essential to address the overall effectiveness of any insomnia medication going forward. The expert panel believes any future insomnia treatment research should include robust, standardized, validated, consistent endpoints focused on daytime functioning. Inclusion of such measures will only serve to improve treatment outcomes for patients going forward. Insomnia does not occur in a vacuum, but rather has many causes and comorbidities. Many insomnia patients are on medications for these comorbid illnesses which may have side effects compromising daytime function. This heterogeneity can obfuscate attempts to understand the impact of insomnia medications on daytime functioning. Lastly, sleep prioritization and good sleep hygiene practices form the basis of healthy sleep and should be implemented broadly to help prevent insomnia from developing in the first place. 

## 5. Conclusions

A thorough review of the available scientific literature showed the evidence did not consistently support the focus statement. Many studies had no measure of daytime functioning, suggesting the field would benefit from consistently including measures of daytime function in future research studies. Novel validated instruments are needed to assess daytime functioning in insomnia disorder. Current field survey participants were predominantly in agreement with the focus statement; however, the expert panelists trended towards strongly disagreeing with the statement post-presentation of the scientific literature. 

## Data Availability

Not applicable.

## Appendix A

Specific search terms used to identify the relevant medical literature include:

- “Sleep Initiation and Maintenance Disorders” [Mesh] and daytime function Filters applied: Meta-Analysis, Systematic Review, 18 results
- Orexin Receptor Antagonists [MeSH Terms] and sleep initiation and maintenance disorders [MeSH Terms], 56 results
- “Sleep Initiation and Maintenance Disorders/drug therapy” [Mesh] AND “Benzodiazepines” [Mesh], Filter Meta-Analysis, Systematic Review, 19 results
- “Sleep Initiation and Maintenance Disorders/drug therapy” [Mesh] and “Hypnotics and Sedatives/therapeutic use” [Mesh], Filters applied: Meta-Analysis, Systematic Review, 29 results
- Sleep Initiation and Maintenance Disorders/drug therapy” [Mesh] and melatonin, Filters applied: Meta-Analysis, Systematic Review, 26 results
- Sleep Initiation and Maintenance Disorders/drug therapy” [Mesh] and diphenhydramine, Filters applied: Meta-Analysis, Systematic Review, 2 results
- “Sleep Initiation and Maintenance Disorders/drug therapy” [Mesh] and “Cognitive Behavioral Therapy” [Mesh] and daytime function, Filters applied: Meta-Analysis, Systematic Review, 3 results
- “Sleep Initiation and Maintenance Disorders/drug therapy” [Mesh] and “Trazodone” Filters applied: Clinical Trial, Meta-Analysis, Randomized Controlled Trial, Systematic Review, 35 results
- “Sleep Initiation and Maintenance Disorders” [Mesh] and Patient Global Impression of Change, limit 10 years, 10 results
- (Insomnia) AND (PROMIS), 30 results
- “Sleep Initiation and Maintenance Disorders/drug therapy” [Mesh] and Daytime Insomnia Symptom Scale, 41 results
- “Sleep Initiation and Maintenance Disorders/drug therapy” [Mesh] and Daytime Consequences of Sleep Questionnaire, limit 10 years, 40 results
- “Sleep Initiation and Maintenance Disorders/drug therapy” [Mesh] and Pittsburgh Insomnia Rating Scale, 28 results
- “Sleep Initiation and Maintenance Disorders/drug therapy” [Mesh] and Profile of Mood States, 13 results
- “Sleep Initiation and Maintenance Disorders/drug therapy” [Mesh] and Sleep Functional Impact Scale, Filter applied: Clinical Trial, Randomized Controlled Trial, 10 results
- “Sleep Initiation and Maintenance Disorders” [Mesh] and Multiple Sleep Latency Test, 1 result
- “Sleep Initiation and Maintenance Disorders” [Mesh] and Maintenance of Wakefulness Test Filters applied: Meta-Analysis, Systematic Review, 9 results
- “Sleep Initiation and Maintenance Disorders/drug therapy” [Mesh] and Maintenance of Wakefulness Test, Filters applied: Clinical Trial, Randomized Controlled Trial, 17 results
- “Sleep Initiation and Maintenance Disorders/drug therapy” [Mesh] and “Pittsburgh Sleep Quality Index” Filters applied: Meta-Analysis, Systematic Review, 11 results
- “Sleep Initiation and Maintenance Disorders/drug therapy” [Mesh] and Epworth Sleepiness Scale, 30 results
- “Sleep Initiation and Maintenance Disorders/drug therapy” [Mesh] and Athens Insomnia Scale, 14 results
- Additional search methods included a manual review of reference lists of relevant articles and an electronic search of ClinicalTrials.gov. From all searches, 42 citations were selected for full review, and of those, 18 were selected for presentation
